# Research on a traditional Chinese medicine case-based question-answering system integrating large language models and knowledge graphs

**DOI:** 10.3389/fmed.2024.1512329

**Published:** 2025-01-07

**Authors:** Yuchen Duan, Qingqing Zhou, Yu Li, Chi Qin, Ziyang Wang, Hongxing Kan, Jili Hu

**Affiliations:** ^1^School of Medical Information Engineering, Anhui University of Chinese Medicine, Hefei, China; ^2^Center for Xin’an Medicine and Modernization of Traditional Chinese Medicine of IHM, Anhui University of Chinese Medicine, Hefei, China

**Keywords:** large language model, knowledge graph, traditional Chinese medicine, question answering system, interdisciplinary research

## Abstract

**Introduction:**

Traditional Chinese Medicine (TCM) case records encapsulate vast clinical experiences and theoretical insights, holding significant research and practical value. However, traditional case studies face challenges such as large data volumes, complex information, and difficulties in efficient retrieval and analysis. This study aimed to address these issues by leveraging modern data techniques to improve access and analysis of TCM case records.

**Methods:**

A total of 679 case records from Wang Zhongqi, a renowned physician of Xin’an Medicine, a branch of TCM, covering 41 diseases, were selected. The study involved four stages: pattern layer construction, knowledge extraction, integration, and data storage and visualization. A large language model (LLM) was employed to automatically extract key entities, including symptoms, pathogenesis, treatment principles, and prescriptions. These were structured into a TCM case knowledge graph.

**Results:**

The LLM successfully identified and extracted relevant entities, which were then organized into relational triples. A TCM case query system based on natural language input was developed. The system’s performance, evaluated using the RAGAS framework, achieved high scores: 0.9375 in faithfulness, 0.9686 in answer relevancy, and 0.9500 in context recall; In human evaluations, the levels of safety and usability are significantly higher than those of LLMs without using RAG.

**Discussion:**

The results demonstrate that integrating LLMs with a knowledge graph significantly enhances the efficiency and accuracy of retrieving TCM case information. This approach could play a crucial role in modernizing TCM research and improving access to clinical insights. Future research may explore expanding the dataset and refining the query system for broader applications.

## 1 Introduction

Traditional Chinese Medicine (TCM) has evolved over thousands of years and represents a distinctive healthcare system that focuses on holistic approaches, prioritizing the balance between body, mind, and spirit. TCM is rooted in ancient philosophies, particularly the concepts of Yin and Yang, as well as the Five Elements theory, which informs its diagnostic and therapeutic approaches. TCM case records, serving as important carriers of TCM knowledge, experience, and wisdom, encompass abundant theoretical knowledge and clinical treatment experiences. These records are crucial for guiding clinical diagnosis and treatment, uncovering therapeutic insights, and advancing the development of TCM (1). However, since TCM case records are frequently documented in classical Chinese, their content is often complex and unstructured.

Traditionally, research on TCM case records has depended on manual reading and analysis to extract and summarize diagnostic and therapeutic patterns. This approach is time-consuming, labor-intensive, and fails to comprehensively mine the valuable information contained in the case records. Consequently, the effective extraction and widespread application of TCM case knowledge have been significantly limited. Consequently, utilizing contemporary technological approaches to automate the extraction and application of TCM case records has emerged as a primary focus in TCM research.

The rapid advancement of Artificial Intelligence (AI), particularly with the emergence of large language models (LLMs), has created new opportunities for the comprehensive mining and structuring of TCM case knowledge. LLMs, such as GPT (2) (Generative Pre-trained Transformer), are advanced AI systems trained on a vast amount of text data, enabling them to process and generate human-like language. These models can extract patterns, standardize terminologies, and analyze unstructured data, making them essential tools for exploring TCM case records and facilitating knowledge integration.

Knowledge Graphs (KGs) are structured semantic databases that organize concepts and demonstrate their interconnections through entities and relationships in a networked form (3). Given the hierarchical richness and complexity inherent in TCM case records, knowledge graphs are particularly well-suited for effectively representing such information.

A question-answering (QA) system refers to a computer program that identifies relevant answers from pre-stored data or information based on user queries and presents them to the user. In contemporary question-answering systems, prevalent approach is standard vector-based RAG (4). Standard vector-based RAG relies on retrieving vectors from the source database and generating responses. Its advantage lies in providing rapid responses by leveraging existing data. However, it may be inadequate when addressing complex problems due to its lack of reasoning capabilities. In contrast, the combination of LLMs and knowledge graphs offers enhanced flexibility and accuracy when addressing complex and ambiguous problems. Language models provide robust natural language processing capabilities, while knowledge graphs contribute rich semantic information and relational reasoning capabilities. This combination not only generates coherent and fluent responses but also facilitates deeper understanding and reasoning in the face of uncertainty. Moreover, due to its structured knowledge management and reasoning capabilities, this approach can provide accurate responses with minimal data, offering advantages in resource utilization and efficiency.

This research aims to explore the role of LLMs in constructing a TCM case knowledge graph and in developing a TCM case-based question-answering system that incorporates LLMs with the knowledge graph. Specifically, in this study, we integrated LLMs with knowledge graphs, enabling the system to understand and process medical queries from users and provide answers grounded in traditional Chinese medical cases. The process includes the automated identification and extraction of key entities, including symptoms, pathogenesis, treatment principles, and medications, from TCM case records through the use of LLMs. The development of the TCM case knowledge graph involves four steps: schema layer construction, knowledge extraction, knowledge fusion, and data storage and visualization. Based on the constructed knowledge graph, we integrate LLMs to develop a QA system for TCM case records. This system enables natural language-based querying of the knowledge graph, providing an effective and practical method for mining and utilizing TCM case knowledge in the realm of TCM research.

## 2 Related work

### 2.1 Research on traditional Chinese medicine case records based on knowledge graphs

Several researchers have employed knowledge graphs to investigate TCM case records. Chen et al. (5) developed a knowledge graph using 120 ancient stroke case records as a data source, achieving “graph-number mutual verification” through semantic data analysis and visualization of the graph. Wang et al. (6) developed a knowledge graph that encapsulates the clinical practices of esteemed TCM practitioners in managing coronary heart disease. This graph facilitates visualization and querying of their diagnostic and therapeutic methods. Yang et al. (7) developed a knowledge graph from TCM clinical data, such as electronic medical records. They introduced a syndrome prediction model (DSDS) based on this knowledge graph, which supports decision-making in syndrome diagnosis. Zhang et al. (8) employed a Naive Bayesian Network and TF-IDF to achieve two-hop path inference within the knowledge graph, thereby providing decision support for TCM clinical practice. Zhao et al. (9) have developed an extensive knowledge graph for diagnosing and treating diabetic kidney disease in the context of TCM. This graph integrates clinical guidelines and real-world data, addressing key clinical challenges and promoting more effective knowledge sharing. These researchers have consistently adopted the knowledge graph approach for the structured processing and visualization of TCM case records, enabling a deeper understanding and analysis of TCM case knowledge. Nonetheless, current research remains constrained in its exploration of knowledge graph applications. Specifically, it has not yet enabled natural language querying of knowledge graphs, implying that direct interaction with the graph using natural language remains unfeasible. Conversely, interaction with the knowledge graph necessitates a certain degree of technical expertise and professional skills, which limits the broader dissemination and application of TCM case knowledge graphs.

### 2.2 Research on traditional Chinese medicine case records based on large language models

The robust natural language understanding and generation capabilities of LLMs have unlocked new possibilities for research and applications across various fields. In the domain of TCM case record studies, LLMs represent a novel and promising tool that may facilitate breakthroughs in the modernization and intelligent development of traditional medicine (10, 11). Li et al. (12) investigated the automated extraction of named entities from case records based on LLMs, creating a tool for structuring medical case texts. Yang et al. (13) examined and described the challenges encountered in TCM intelligent diagnosis and treatment. They proposed a research methodology that utilizes LLMs, encompassing corpus preparation, knowledge representation, instruction fine-tuning, and reinforcement learning. Zhang et al. (14) investigated techniques for utilizing LLMs in the development of TCM knowledge graphs, thereby improving the representation, storage, and utilization of TCM knowledge. Wang et al. (15) employed medical knowledge graphs and literature to fine-tune the base model, resulting in ShenNong-TCM, which enhanced the model’s question-answering capabilities in the medical field. Kang et al. (16) created a multi-scenario instruction dataset based on TCM gynecology prescription data, resulting in CMLM-ZhongJing, which augmented the model’s reasoning abilities in TCM prescription data and diagnostic logic. Zhang et al. (17) integrated TCM textbooks and other corpora into the Ziya-LLaMA-13B-V1 model, performing pre-training and instruction fine-tuning on ancient TCM texts to create HuangDi, which provided the model with the ability to answer questions on ancient TCM knowledge. These studies illustrate the significant potential of LLMs in TCM case record research. However, several challenges and concerns still emerge in practical applications. First, the challenge of hallucination (18), wherein the model generates inaccurate or unreliable outputs. Second, the absence of natural language dialogue-based querying for TCM knowledge graphs. Therefore, optimizing LLMs to ensure the accuracy of output results and developing more sophisticated dialogue systems to enhance the application of TCM knowledge graphs are crucial directions for improving the effectiveness of these applications.

### 2.3 Research combining knowledge graphs and large language models

The integration of LLMs with KGs has been shown to be an effective strategy for reducing hallucinations in LLMs. Guan et al. (19) introduced the KGR algorithm, allowing LLMs to extract, verify, and refine factual statements by utilizing the knowledge stored in KGs. This method directly addresses the hallucination issue in LLMs. Sun et al. (20) proposed the ToG method, establishing a new paradigm for LLM-KG integration referred to as “LLM ⊗ KG.” In this approach, LLMs act as agents exploring relevant entities and relationships within the KG, performing reasoning based on the retrieved knowledge, thereby enhancing knowledge traceability and modifiability in LLMs. Yang et al. (21) developed a KG-enhanced large language model (KGLLM), providing a solution to improve LLMs’ factual reasoning capabilities. Carlos et al. (22) successfully demonstrated interpretable reasoning by combining KGs and LLMs. Zhang et al. (23) designed a question-answering system grounded in LLMs and KGs, utilizing TCM prescriptions as an example, which facilitates information filtering, professional question-answering, and the capabilities of extraction and transformation. These studies indicate that KGs, as a structured form of knowledge representation, can provide models with enhanced and more accurate contextual information. By combining LLMs with KGs, models can access comprehensive and precise knowledge input, thereby reducing biases and inaccuracies in the generative process. Therefore, the integration of LLMs with KGs holds promise for mitigating hallucinations in LLMs and provides more reliable and effective support for TCM case study research. Although these studies demonstrate the potential of combining LLMs and KGs, the exploration of their in-depth applications in the specialized domain of TCM case studies remains limited. To address this gap, this study proposes a method for constructing a TCM case knowledge graph aided by LLMs and develops a question-answering system for TCM case studies by integrating LLMs and KGs, specifically focusing on their application within the TCM case study domain.

## 3 Materials and methods

### 3.1 Overview of the QA system framework

**Figure 1:**
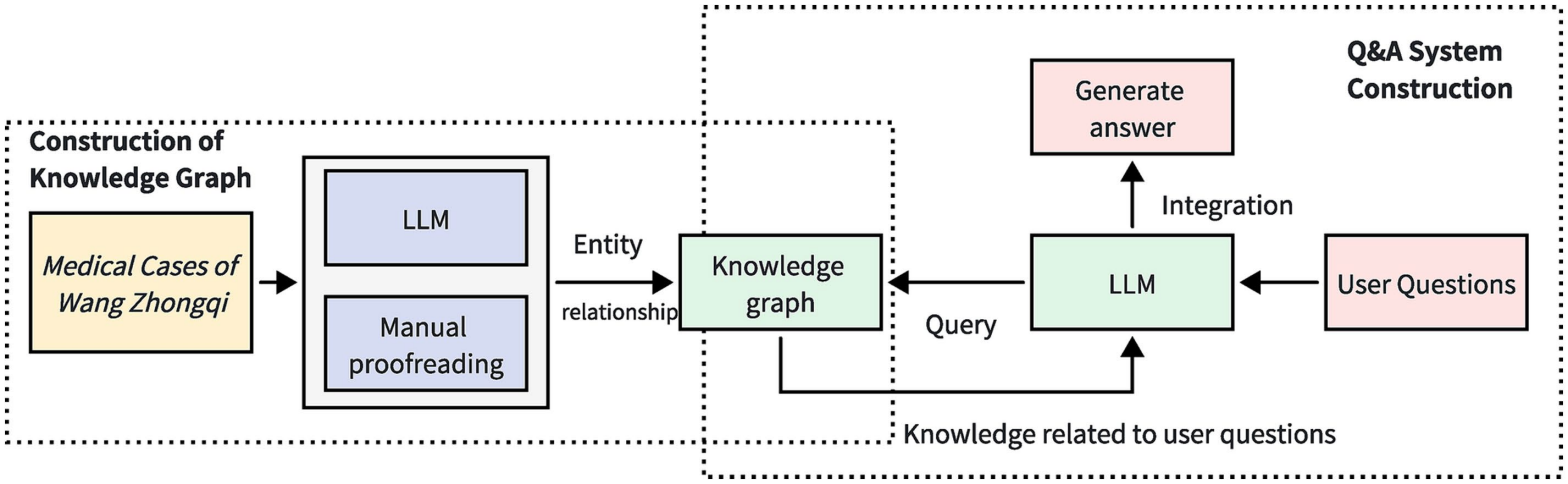
The overall framework of Traditional Chinese Medicine Case Q&A System Combining Big Language Model and Knowledge Graph. By utilizing large language models and manual proofreading, the *Medical Cases of Wang Zhongqi* are organized into a knowledge graph. When a user submits a question, the large language model recognizes the query content from the user’s inquiry and conducts a search within the knowledge graph. Relevant information pertaining to the user’s question is delivered to the large language model, which then integrates this information to generate a response.

The overall structure of the TCM case question-answering system, which incorporates LLMs along with knowledge graphs, is illustrated in Figure 1. This system structure primarily comprises two key components: knowledge graph construction and question-answering system development. In the knowledge graph construction phase, this study utilizes a hybrid approach of LLMs and manual verification to develop the TCM case knowledge graph. Based on the constructed knowledge graph, a TCM case question-answering system is subsequently developed. In the question-answering system development phase, the fusion of LLMs and knowledge graphs exploits the natural language processing capabilities of the LLMs to interpret user inquiries. By querying the knowledge graph, relevant information related to the user’s inquiries is extracted. Finally, the acquired knowledge is synthesized and articulated, and presented to the user in a natural language format, thereby enhancing the facilitation of natural language queries within the TCM case knowledge graph.

### 3.2 Methods for knowledge graph construction

**Figure 2:**
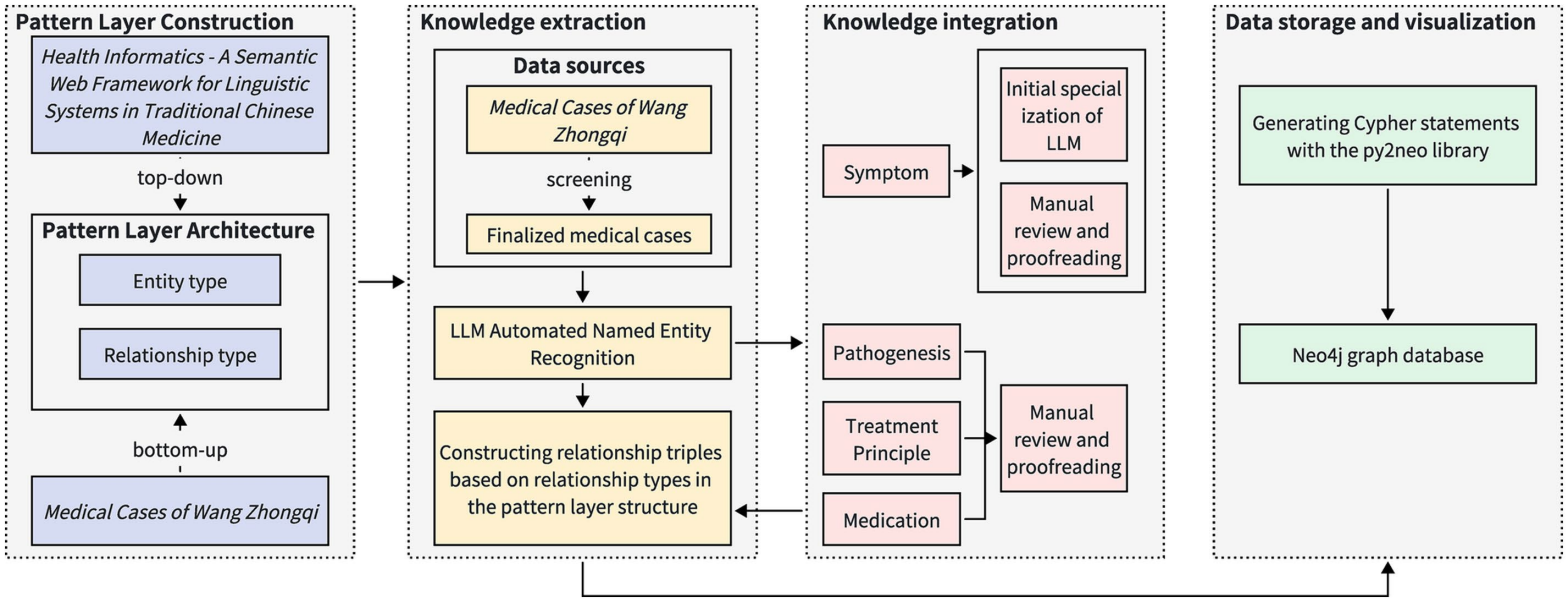
Process diagram of knowledge graph construction method. The construction process of the pattern layer is based on the national standards of Traditional Chinese Medicine and the specific structure of medical cases, which encompasses the definition of entity types and relationship types. The data extraction process involves selecting appropriate medical cases from the *Medical Cases of Wang Zhongqi*, followed by automated entity recognition using a large-scale language model. Subsequently, knowledge fusion is conducted individually for different entity types, resulting in the formation of relationship triples. The data storage and visualization process employs Cypher queries to store the data in the Neo4j graph database.

During the process of constructing a knowledge graph, a structured method is applied systematically (24). This methodology comprises four steps: schema layer construction, knowledge extraction, knowledge fusion, and data storage and visualization. Knowledge extraction entails recognizing named entities and their relationships. Figure 2 depicts the process for constructing the knowledge graph via a flowchart.

#### 3.2.1 Data sources

The main data source for constructing the knowledge graph is the *Medical Cases of Wang Zhongqi* (25). This book records the clinical cases of Wang Zhongqi, a renowned modern Chinese physician, from 1923 to 1935. Assembled and carefully selected by his students over a period exceeding two decades, the book distills the essence of the original medical cases, thoroughly demonstrating Wang Zhongqi’s medical expertise. Issued by a reputable publishing house, the book exhibits a high degree of accuracy and authority. We inputted and processed the data from the manuscript and compiled it into an Excel spreadsheet, obtaining a total of 1,573 medical cases. To ensure the precision of the knowledge graph, our study only included medical cases that explicitly contained all entity information, such as disease names, symptoms, pathogenesis, treatment principles, and medications. Medical cases missing any entity information were excluded from the dataset. Following screening, a total of 679 cases were included, encompassing 41 distinct diseases. The distribution of diseases within the medical cases is illustrated in Table 1.

**Table 1:** Distribution of diseases included in medical records.

Disease name	Frequency	Disease name	Frequency	Disease name	Frequency
Abdominal Pain	56	Liver Yang Rising and Liver Wind	15	Flooding and Spotting	9
Seasonal Illness	47	Abdominal Masses and Lumps	15	Leucorrhea	9
Diarrhea	38	Lin Syndrome and Turbid Urine	15	Nosebleed	9
Hemoptysis	37	Nocturnal Emission and Impotence	15	Constipation	8
Damp-Heat Syndrome	36	Various Orifices	15	Postpartum	7
Dysentery	30	Jaundice	14	External Syndrome	7
Irregular Menstruation	28	Vomiting and Diarrhea	14	Deficiency Fatigue	7
Abdominal Distension	24	Vomiting	13	Intestinal Parasites	5
Cough	23	Throat	13	Edema	5
Stroke	23	Nourishing Tonic Pastes, Pills, and Powders	13	Phlegm-Fluid Retention	5
Spleen and Stomach Disorders	19	Tuberculosis	12	Lung Abscess	3
Atrophy	18	Depressive Disorder	12	Sweating Disorder	2
Asthma	18	Insomnia	11	Diabetes	2
Blood Stasis	16	Malaria Pathogen	11		
Total					679

#### 3.2.2 Schema layer construction

The development of the schema layer (26) entails the definition of the information structure and data model within the knowledge graph, incorporating the types of entities and the relationships between them.

To ensure the accuracy and coherence of the schema layer development, we first consult the semantic types and semantic relationships articulated in the “*Health Informatics—Semantic Network Framework of Traditional Chinese Medicine Language System*” (27) and employ a top-down approach to design the schema layer structure. Subsequently, in alignment with the specific structural characteristics of the *Medical Cases of Wang Zhongqi,* we utilize a bottom-up approach to refine the schema layer structure, thereby enhancing its accuracy in reflecting the content of the medical cases.

The entity types in the knowledge graph are delineated in Table 2, while the relationship types are illustrated in Table 3.

**Table 2:** Entity type.

Entity type	Definition
Disease name	The name of a pathological process characterized by the conflict between pathogenic factors and the body’s normal defenses, such as dysentery and asthma.
Symptom	The abnormal state exhibited by the body due to disease, encompassing various anomalous sensations experienced by the patient as well as abnormal presentations perceived by the physician’s sensory organs, such as cough and dizziness.
Pathogenesis	The study of the mechanisms underlying the development and progression of diseases based on foundational theories of Traditional Chinese Medicine (TCM), such as lung qi rebelliousness and liver yang hyperactivity.
Treatment Principle	A comprehensive analysis of disease based on holistic concepts and differential diagnosis, utilizing objective data collected through the four diagnostic methods to formulate treatment rules tailored to different pathogenesis, such as dispersing wind and relieving the exterior or soothing the liver and resolving depression.
Medication	Medicines used for disease prevention, treatment, and healthcare, guided by TCM theories and clinical experience, such as Poria (Fu Ling) and Astragalus (Huang Qi).

**Table 3:** Relationship type.

Relationship	Subject	Object	Example
Belongs to	Symptom	Disease Name	Hemiplegia—belongs to -> Stroke
Causes	Pathogenesis	Symptom	Stomach Qi Rebellion—causes -> Hiccup
Follows	Pathogenesis	Treatment Principle	Yin Deficiency with Hyperactivity of Fire—follows -> Nourish Yin and Reduce Fire
Recommends	Treatment Principle	Medication	Dispel Exterior and Ventilate the Lung—recommends -> Mulberry Leaf
Applies	Symptom	Treatment Principle	Cough—applies -> Dispel Exterior and Ventilate the Lung
Treats	Medication	Pathogenesis	Angelica dahurica (Bai Zhi)—treats -> Defensive Qi Deficiency
Treats	Medication	Symptom	Mint (Bo He)—treats -> Head Distention and Pain

#### 3.2.3 Knowledge extraction

Knowledge extraction (28) is defined as the process of transforming raw data into standardized categories, with the primary goal of identifying entities and the relationships among them from various data sources. In this study, knowledge extraction is comprised of two steps: named entity recognition and relationship extraction.

##### 3.2.3.1 Named entity recognition

Named Entity Recognition (NER) is a critical component of natural language processing. Its goal is to automatically identify and classify entities with specific meanings from text, forming the basis for knowledge graph construction. In this study, the classification of disease names is based on the chapters of *Medical Cases of Wang Zhongqi,* and the entities targeted for extraction from these medical cases include symptoms, pathogenesis, treatment principles, and medicinal usage.

This research employs LLMs to automate the named entity recognition process, utilizing the GLM-3-Turbo API (29) in conjunction with few-shot learning (30) strategies. The goal is to overcome the practical constraint of limited annotated data resources in the field of TCM, capitalizing on the generalization capabilities of LLMs to accurately identify key entity information in medical case records.

Using symptom entity recognition as an example, the prompt design is detailed in Table 4. The System section includes the task description, output format requirements, and specific examples utilized for few-shot learning, while the Human section provides the input medical case text that requires named entity recognition. To ensure the accuracy of NER achieved by the LLM, three typical examples were constructed in the few-shot learning section:

**Table 4:** Prompt of symptom entity recognition.

System: 你的任务是提取出文本中的症状，用列表的格式列出，若文本中无症状请输出“NaN”。以下是一些例子: Input: 腰疽愈后，肾伤未复，风邪易乘虚而袭，身热形寒，头脑胀痛眩晕，项背肩胛酸胀，脉浮而濡，舌干而燥。 Output: [‘身热形寒’, ‘头脑胀痛眩晕’, ‘项背肩胛酸胀’, ‘脉浮而濡’, ‘舌干而燥’] Input: 再以宣湿除陈，以醒胃气，调理尚须从缓也。 Output: NaN Input: 燥屎已下，郁热下行，颧红、唇绛、舌赤均已退淡，芒刺亦软，惟午后稍觉烦躁，入夜犹欠清爽。 Output: [‘惟午后稍觉烦躁’, ‘入夜犹欠清爽’] Human: 使用给出的格式提取以下文本中的信息。 Input: {中医医案} Output:	System: Your task is to extract symptoms from the text and list them in a format like a list. If there are no symptoms, output ‘NaN’. Here are some examples: Input: After recovering from a lumbar abscess, kidney injury remains unresolved, making it easy for wind pathogens to take advantage of the deficiency. The patient experiences body heat with aversion to cold, head distention with dizziness, aching and distension in the nape and scapula, a floating and soft pulse, and a dry and parched tongue. Output: [‘Body heat with aversion to cold’, ‘Head distention with dizziness’, ‘Aching and distension in the nape and scapula’, ‘Floating and soft pulse’, ‘Dry and parched tongue’] Input: Followed by dispelling dampness and eliminating stagnation to awaken stomach qi, the treatment needs to be adjusted slowly. Output: NaN Input: Dry stools have been passed, the heat has descended, and the red cheeks, crimson lips, and red tongue have faded. The prickles on the tongue have softened, but the patient still feels some restlessness in the afternoon and is not entirely comfortable at night. Output: [‘Restlessness in the afternoon’, ‘Not entirely comfortable at night’] Human: Extract information from the following text using the given format. Input: {TCM medical case} Output:

A sentence that explicitly provides symptom descriptions. This example ensures that the LLM can learn and grasp the main characteristics of symptom expressions within their contextual environment, enhancing the model’s sensitivity to symptom entities.

A sentence that does not include symptom information. This serves as a counterexample, aiding the model in learning the correct response mechanism in the absence of target entities, thereby reducing the likelihood of incorrect recognition.

A sentence containing modifiers related to symptom alleviation. This is intended to strengthen the model’s ability to handle complex expressions, enabling accurate identification of symptom entities across various descriptive forms and enhancing both comprehensiveness and robustness in recognition.

##### 3.2.3.2 Relation extraction

The extraction of entity relations is an essential task in the field of information retrieval. It involves the identification and extraction of specified relationships between entities from unstructured text, which relies on entity recognition. These relationships are typically expressed as relational triples $\langle e_{1},r,e_{2}\rangle$ , where $e_{1}$ and $e_{2}$ each represent entities, and the connection r between them is defined by a predefined set of relationships $R\left\{r_{1},r_{2},r_{3},\ldots ,r_{i}\right\}$ . The goal of relation extraction is to obtain these relational triples $\langle e_{1},r,e_{2}\rangle$ from natural language text, which helps acquire relevant information from the text.

To reflect the principles of TCM and to reveal the complex relationships between entities such as disease names, symptoms, medications, pathogenesis, and treatment principles, the extracted entities need to be efficiently combined according to the relationship types established in the schema construction phase. This will facilitate the formation of TCM-specific relational triples. In this study, the extracted entities are recorded in an Excel spreadsheet, and the pandas library^1^ is utilized to read and combine the triples.

#### 3.2.4 Knowledge fusion

Knowledge fusion refers to the process of unifying and integrating heterogeneous data extracted from various types of knowledge. Due to the personalized nature of medical case records, the terminology within these records often displays non-standardized characteristics, failing to comply with uniform expression guidelines. To ensure the standardization of entities, it is necessary to systematically normalize the extracted entities, with the goal of eliminating the variations in personalized expressions in medical case records and to standardize the terminology.

**Figure 3:**
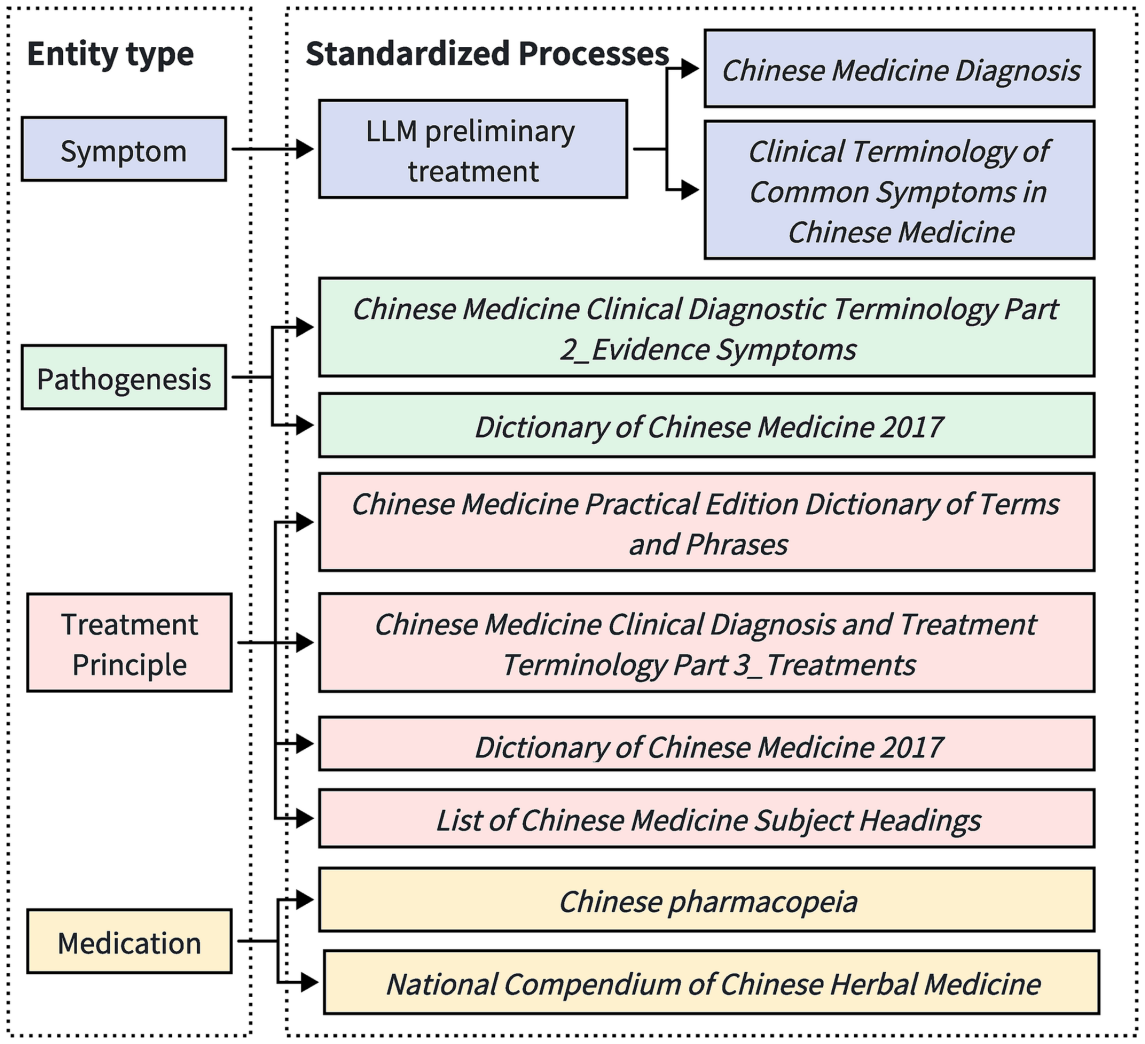
Process flow diagram for standardizing different entities. Given the diversity and complexity of symptom descriptions, symptom entities are subjected to initial processing by a large-scale model, followed by subsequent manual verification based on standards issued by recognized organizations. The entities pertaining to pathogenesis, treatment principles, and medication are standardized directly according to the regulations published by these recognized bodies.

This study adopts a LLM-assisted approach, combined with manual proofreading, to unify and normalize the entities extracted during the knowledge extraction process, as illustrated in Figure 3.

For symptom entities, given the diversity and complexity of symptom descriptions, the LLM is initially used to perform a preliminary professional processing of the symptom entities. This aims to capture and standardize the core symptoms expressed in multiple forms. Subsequently, manual review and proofreading are conducted to ensure the consistency and accuracy of the symptom terminology. Our approach to manual proofreading includes inviting two researchers with backgrounds in traditional Chinese medicine to independently review the extracted entities. To enhance accuracy, we employ a cross-proofreading method, where each researcher’s corrections are cross-verified by the other. This collaborative process ensures high standards of precision and consistency in the terminology used. The manual proofreading process follows the guidelines from the *Normative Terminology for Common Symptoms in Traditional Chinese Medicine* and *Diagnostics of Traditional Chinese Medicine*, striving for simplicity and generality in linguistic expression. For example, non-standardized colloquial descriptions such as “slight abdominal bloating” are normalized to “abdominal distension”; compound symptoms, such as “swelling in the neck and below the ear,” are split into “neck swelling” and “subaural swelling.” However, for entities that describe the nature of symptoms, to preserve the integrity of the symptom description, no splitting is performed, such as keeping “head distension and pain” as “head distension and pain.”

For pathomechanism entities, standardization is conducted based on the *Clinical Terminology of Traditional Chinese Medicine—Part 2: Syndrome* and the *2017 Dictionary of Traditional Chinese Medicine*. For therapeutic principle entities, normalization adheres to the *Clinical Terminology of Traditional Chinese Medicine—Part 3: Therapeutic Methods*, *2017 Dictionary of Traditional Chinese Medicine*, *Practical Dictionary of Traditional Chinese Medicine Terminology*, and the *Subject Headings of Chinese Medicine*. For medication entities, standardization is conducted according to the *Chinese Pharmacopoeia* and the *Compendium of Chinese Herbal Medicine*.

#### 3.2.5 Data storage and visualization

This study presents the knowledge of TCM medical cases in the form of a knowledge graph, using triples $\left\{\left(h_{i},r_{i},t_{i}\right)|i\in \left[1,N\right]\right\}$ to define knowledge, where each $\left(h_{i},r_{i},t_{i}\right)$ corresponds to a head entity, a relationship, and a tail entity. 𝑁 denotes the quantity of triples in the knowledge graph. Given a knowledge graph, the triples within the graph can be encoded into Equation 1.


(1) $$S=\left[\left[s\right],h_{1},r_{1},t_{1},\left[e\right],h_{2},r_{2},t_{2},\left[e\right],\ldots ,h_{N},r_{N},t_{N},\left[e\right]\right]$$

**Figure 4:**
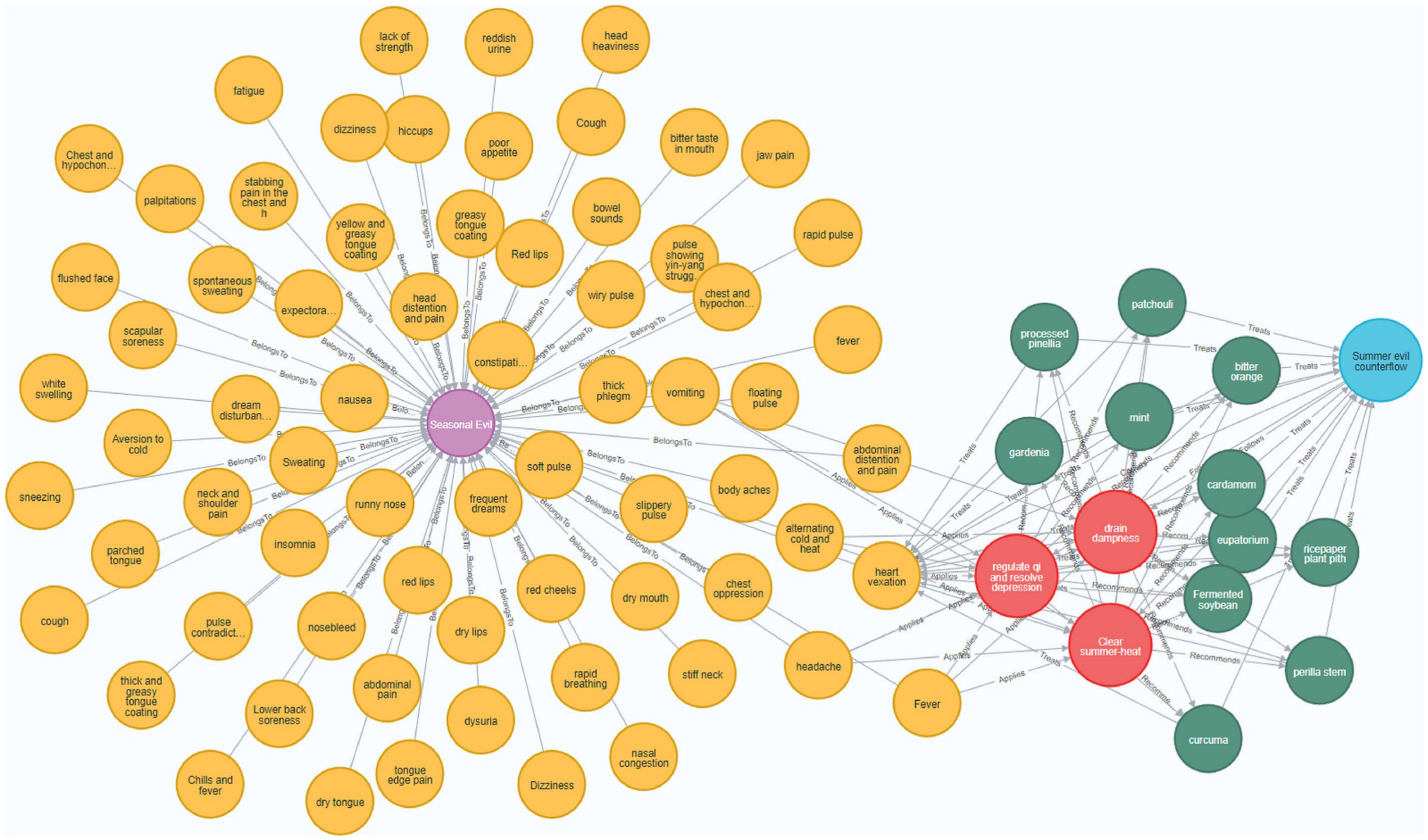
Visualization of the *Medical Cases of Wang Zhongqi* Knowledge Graph. In this figure, the purple nodes represent disease names, yellow nodes represent symptoms, blue nodes represent pathogenesis, red nodes represent treatment principles, and green nodes represent medications. The relationships between these entities are indicated as follows: disease names are connected to symptoms by the “BelongsTo” relationship, symptoms are connected to treatment principles by the “Applies” relationship, symptoms are linked to pathogenesis by the “Causes” relationship, treatment principles are connected to medications by the “Recommends” relationship, pathogenesis is connected to treatment principles by the “Follows” relationship, medications are linked to symptoms by the “Treats” relationship, and medications are also connected to pathogenesis by the “Treats” relationship.

The Neo4j graph database (31), due to its adaptive data model, highly efficient query performance, and facilitated scalability and integration, has become a widely used graph database for constructing knowledge graphs. This study utilizes the Neo4j graph database to store and visualize the knowledge graph, leveraging the Py2neo library^2^ to create Cypher queries for building the graph. A partial view of the developed knowledge graph is presented in Figure 4.

In the graph, the nodes depict the entities within the triples, while the edges illustrate the relationships in the triples. A relationship between two nodes is represented by an edge directed from one node to the other, with the edge’s direction indicating a relationship from the subject to the object.

### 3.3 Methods for constructing a QA system

**Figure 5:**
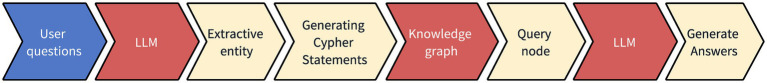
Q&A system construction flowchart. After the user has submitted a question, a large language model is invoked to perform extractive entity recognition and generate Cypher statements. This process queries the nodes in the knowledge graph, and based on the information retrieved from the knowledge graph, an answer is generated.

Drawing upon the constructed knowledge graph, the integration of LLMs with the knowledge graph seeks to facilitate natural language queries for the TCM case knowledge graph. The method for constructing the question-answering system consists of four primary steps: entity extraction, Cypher query generation, node querying, and response generation. The flowchart depicting the construction of the question-answering system is presented in Figure 5.

Utilizing the Langchain library^3^ and interfacing with the GPT-3.5-Turbo API, the connection between the LLM and the knowledge graph is established, allowing the language model to interpret the structure and content of the knowledge graph.

#### 3.3.1 Extracting entities

When a user submits a query to a LLM, the model employs its natural language processing capabilities to analyze the query and identify key entities within it, such as diseases, symptoms, or traditional Chinese medicine. The purpose of this step is to accurately understand the user’s question, which acts as the foundation for subsequent query processes.

#### 3.3.2 Generating cypher statements

Based on the extracted entities, relevant Cypher queries are generated by the LLM. Due to the inherent instability in the results generated by the LLM, a consistent structure is employed to ensure the accuracy of the generated Cypher queries. The model populates the extracted entities within the predefined structure to produce the Cypher queries.

#### 3.3.3 Querying nodes

The generated Cypher queries are then sent to the knowledge graph for execution, returning the entity nodes pertinent to the user’s query, along with the relationships between these entities.

#### 3.3.4 Generating answers

**Figure 6:**
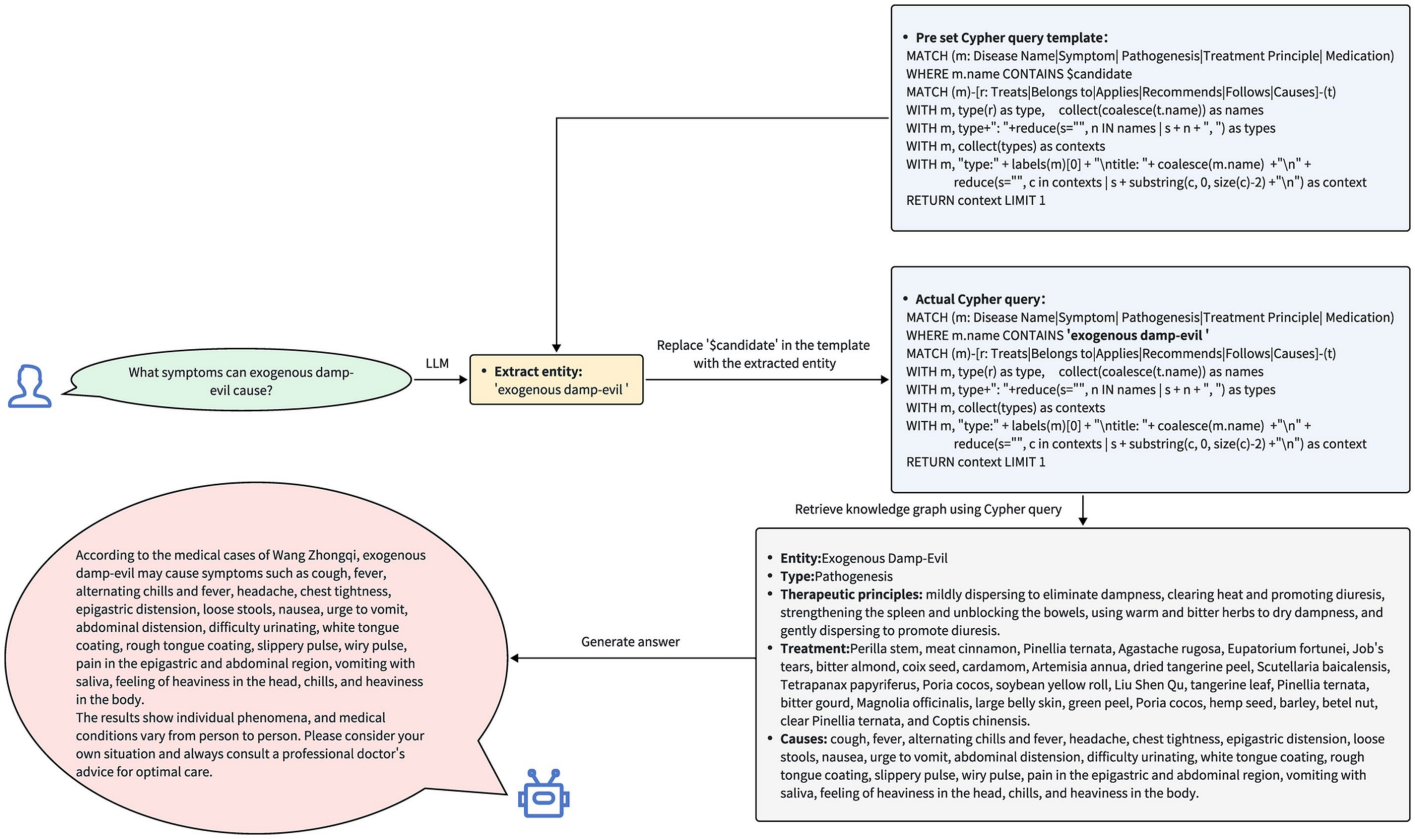
The query process and responses of the question-answering system. Upon receiving the user’s query, the system first extracts entities related to Traditional Chinese Medicine from the question. The system then inserts these extracted entities into a pre-defined Cypher query template and uses the populated Cypher query to search for these entities, their related entities, and their interrelationships within the knowledge graph. Finally, based on the retrieved data, the system generates and provides a response to the user’s query.

Based on the results returned from the knowledge graph, the system formulates a response to be presented to the user. This procedure ensures the precision and pertinence of the response, as illustrated in Figure 6.

## 4 Results

To evaluate the capabilities of named entity recognition (NER) among various LLMs in the context of constructing knowledge graphs and their performance in response generation through integration with knowledge graphs, a comparative experiment was conducted on five representative models: GPT-3.5-Turbo, GLM-3-Turbo, Qwen-Turbo, Spark3.5 Max, and Moonshot-V1-8K. We utilized APIs to interact with these models, enabling efficient testing and comparison of their functionalities.

As part of OpenAI’s GPT series, GPT-3.5-Turbo is noted for its robust text generation and comprehension capabilities, where robust text generation refers to the model’s ability to generate text that is coherent, contextually relevant, and grammatically accurate across a broad range of topics. This strength allows it to excel in various natural language processing tasks. This model has demonstrated strong performance in understanding complex inputs and generating meaningful outputs. Qwen-Turbo not only demonstrates excellence in text generation but also exhibits strong proficiency in integrating structured knowledge. GLM-3-Turbo has been specifically optimized for Chinese-language environments, making it particularly skilled at understanding and generating Chinese text. Spark3.5 Max demonstrates exceptional performance in downstream tasks that require a profound understanding and application of extensive background knowledge. Finally, Moonshot-V1-8K is noted for its high level of innovation and is particularly proficient in handling long-sequence texts.

### 4.1 Evaluation of named entity recognition

In Named Entity Recognition (NER) tasks, key metrics for evaluating model performance include precision, recall, and the F1 score.

Precision represents the ratio of correctly identified entities to the total entities predicted by the model, as detailed in Equation 2.


(2) $$\mathrm{Precision}=\frac{TP}{TP+FP}$$

Recall assesses the ratio of correctly identified entities compared to all actual entities in the dataset, as outlined in Equation 3.


(3) $$\mathrm{Recall}=\frac{TP}{TP+FN}$$

The F1 score is a widely used statistical metric for assessing the performance of binary classification models. It is calculated as the harmonic mean of precision and recall, as shown in Equation 4.


(4) $$F1=2\times \frac{\mathrm{Precision}\times \mathrm{Recall}}{\mathrm{Precision}+\mathrm{Recall}}$$

TP (True Positive) refers to the number of entities accurately identified by the model. FP (False Positive) refers to instances where the model incorrectly classifies non-entities as entities. FN (False Negative) denotes the number of actual entities that the model fails to accurately identify.

All models were assessed on the same test set, which was randomly selected from the *Medical Cases of Wang Zhongqi*. The test set contains 200 annotated sentences, covering entity types such as symptoms, pathogenesis, and treatment principles.

The evaluation results are summarized in Table 5.

**Table 5:** Named entity recognition evaluation results.

	Precision	Recall	F1 scores
GPT-3.5-Turbo	0.4834	0.8989	0.6287
GLM-3-Turbo	**0.9462**	**0.9778**	**0.9617**
Spark3.5 Max	0.8173	0.9200	0.7892
Qwen-Turbo	0.6335	0.8883	0.7395
Moonshot-V1-8 K	0.9086	0.8833	0.8958

Bold values indicate superior performance.

The evaluation results indicate that the GLM-3-Turbo model exhibited exceptional overall performance, with the highest precision, recall, and F1 scores, which were 0.9462, 0.9778, and 0.9617, respectively. This suggests that the model not only accurately identifies entities but also efficiently minimizes false positives. Although the GPT-3.5-Turbo model performed well in terms of recall, its precision was comparatively low, resulting in an F1 score of merely 0.6287. This implies that while the model can recognize most entities, it also has a considerable false positive rate. Both the Spark3.5 Max and Qwen-Turbo models exhibited satisfactory recall, but their precision was notably lower, indicating that these models have some capability in entity detection but still necessitate improvement in minimizing false positives. The Moonshot-V1-8K model achieved a favorable balance between precision and recall, resulting in a comparatively high F1 score.

Among all the evaluated models, the GLM-3-Turbo model exhibited the highest overall performance and was therefore chosen for constructing the TCM case knowledge graph.

### 4.2 Evaluation of generated responses

The question-answering system that integrates LLMs with knowledge graphs can be viewed as a Retrieval-Augmented Generation (RAG) process, where the knowledge graph serves as an external database. Therefore, the RAGAS framework is chosen for evaluation, aiming to objectively assess the system’s information retrieval and generation capabilities.

**Figure 7:**
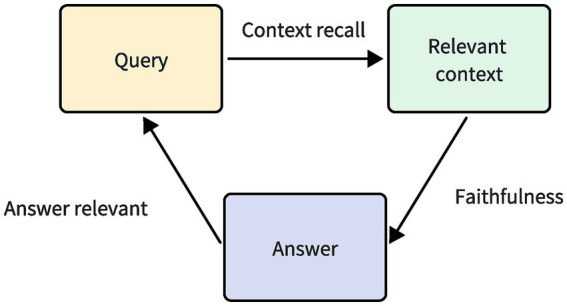
RAGAS evaluation framework. It evaluates the effectiveness of RAG by measuring the relevance between the query, answer, and relevant context.

RAGAS (32) is an automated evaluation framework for RAG processes, which assesses the effectiveness of RAG by analyzing the correlation among the three key elements: the input query, the retrieved context, and the response generated by the LLM. Its evaluation metrics include faithfulness, answer relevance, and context recall, as illustrated in Figure 7.

Faithfulness measures whether the answer is based on the provided context. First, a set of statements $S\left(a\left(q\right)\right)$ is extracted using a LLM, which then assesses whether each statement $s_{i}$ can be deduced from $c\left(q\right)$ . The final faithfulness score F is calculated as shown in Equation 5, where |V| represents the number of statements supported by the LLM, and |S| represents the total number of statements.


(5) $$F=\frac{\left|V\right|}{\left|S\right|}$$

Answer relevance is an indicator of the correspondence between the generated answer and the query. The LLM generates n potential questions $q_{i}$ based on the given answer, and for each question $q_{i}$ , it computes the similarity $sim\left(q,q_{i}\right)$ to the original query q through cosine similarity of the embeddings. The answer relevance score AR for the question q is calculated as shown in Equation 6.


(6) $$AR=\frac{1}{n}\overset{n}{\underset{i=1}{\sum }}sim\left(q,q_{i}\right)$$

Context recall measures the degree of consistency between the retrieved context and the annotated answer. The formulation is presented in Equation 7.


(7) $$\mathrm{context recall}=\frac{\left|\begin{array}{l} \mathrm{Ground truth sentences that}\;\mathrm{can}\;\mathrm{be}\;\mathrm{attributed}\;\\ \mathrm{to context} \end{array}\right|}{\left|\begin{array}{l} \mathrm{Number of sentences in Ground}\;\\ \mathrm{truth sentences} \end{array}\right|}$$

To comprehensively evaluate the performance of the TCM case-based question-answering system, 20 evaluation questions were designed, covering core dimensions such as diseases, symptoms, pathogenesis, treatment principles, and medication. The evaluation questions, retrieved contexts, responses generated by the question-answering system, and the correct answers to the evaluation questions were compiled into a CSV file to construct the evaluation dataset. The composition of this dataset is shown in Table 6.

**Table 6:** Evaluate dataset structure.

	Definition
Question	An evaluative query posed for assessment purposes.
Answer	The response generated by the question-and-answer system.
Contexts	Contextual information retrieved from the knowledge graph.
Ground truths	The correct answers corresponding to the evaluative queries.

The evaluation results of RAGAS are presented in Table 7.

**Table 7:** RAGAS evaluation results.

	Faithfulness	Answer relevance	Context recall
GPT-3.5-Turbo	**0.9375**	**0.9686**	**0.9500**
GLM-3-Turbo	0.8289	0.9036	0.9083
Spark3.5 Max	0.6066	0.9477	0.9344
Qwen-Turbo	0.5687	0.8099	0.9474
Moonshot-V1-8K	0.7708	0.9235	0.9357

Bold values indicate superior performance.

The evaluation results indicate that the GPT-3.5-Turbo model achieved the highest scores in three dimensions: fidelity, answer relevance, and context recall, with scores of 0.9375, 0.9686, and 0.9500, respectively. This suggests its outstanding performance in terms of the accuracy and relevance of generated answers, as well as its ability to effectively integrate contextual information. The GLM-3-Turbo model exhibited balanced performance, with context recall comparable to that of GPT-3.5-Turbo. Spark3.5 Max excelled in answer relevance but exhibited lower fidelity. Qwen-Turbo demonstrated suboptimal performance in both answer relevance and fidelity metrics. The overall performance of the Moonshot-V1-8K model was considered moderate.

The GPT-3.5-Turbo model exhibited significant advantages in the RAGAS evaluation; thus, it was selected for integrating LLMs with knowledge graphs and generating responses.

In summary, the integration of LLMs with knowledge graphs in the TCM case system demonstrated superior performance in terms of answer accuracy, question-answer relevance, and context application, enabling efficient and accurate querying of the TCM case knowledge graph.

### 4.3 Manual evaluation

To validate the safety, accuracy, and professionalism of the question-answering system, this study employed the SUS (15) (Safety, Usability, Smoothness) evaluation method. A comparative evaluation will be conducted between a question-answering system integrated with LLM and knowledge graphs, and a LLM without RAG technology. The SUS evaluation method consists of three dimensions: safety, usability, and smoothness. Safety evaluates whether the model-generated content could mislead users and pose potential health risks; usability assesses whether the content meets professional knowledge requirements; smoothness examines the response stability and naturalness of the model. SUS employs a three-point scoring system, with scores ranging from 1 (unacceptable) to 3 (good), where 2 indicates acceptable performance. To evaluate the model’s performance, five evaluators with backgrounds in traditional Chinese medicine were invited to score 20 randomly selected diagnostic questions related to traditional Chinese medicine. The evaluators analyzed the LLMs’ responses to determine whether they posed a health risk to users, whether they demonstrated professional Chinese medicine knowledge, and whether they were suitable for public use. Based on the three dimensions of the SUS evaluation, the model’s question-answering performance was analyzed and scored in terms of safety, usability, and smoothness. Table 8 presents the evaluation results.

**Table 8:** SUS evaluation results.

	Safety	Usability	Smoothness
GPT-3.5-Turbo	1.64	1.57	2.2
GLM-3-Turbo	1.57	1.56	2.13
Spark3.5 Max	1.67	1.65	2.21
Qwen-Turbo	1.74	1.67	2.17
Moonshot-V1-8K	1.67	1.55	2.17
Our QA system	**2.868**	**2.848**	**2.98**

Bold values indicate superior performance.

According to the SUS evaluation results, our Q&A system performed excellently in all dimensions, especially excelling in safety and usability, fully demonstrating its reliability and professionalism in providing traditional Chinese medicine health consultation services to users. Specifically, the system excels in safety and usability, effectively preventing the generation of erroneous or misleading health advice, ensuring that users are not exposed to potential medical risks. This is something traditional LLMs (e.g., GPT-3.5-Turbo and GLM-3-Turbo) cannot achieve, as these models typically lack specialized knowledge in the field of traditional Chinese medicine and are prone to generating inaccurate answers in this area.

Although other existing LLMs perform excellently in smoothness, generating relatively natural and coherent language, they still have significant room for improvement in terms of safety and usability. This is because the answers from these general-purpose LLMs may lack specialized knowledge in traditional Chinese medicine, leading them to fail in providing sufficiently accurate or personalized answers when faced with Chinese medicine diagnostic and treatment issues. Moreover, since they do not integrate the knowledge graph of traditional Chinese medicine case studies, they may struggle to access the clinical experience of real-world Chinese medicine practitioners, thus affecting the professionalism of the Q&A.

In contrast, our system combines LLMs with a knowledge graph, particularly in utilizing the Chinese medicine case knowledge graph to enhance the model’s professionalism. The knowledge graph supplements the language model’s lack of depth in knowledge, and by using the relationships between symptoms, disease names, treatment principles, pathogenesis, and medications in Chinese medicine case studies, it significantly enhances the accuracy and professionalism of the system’s responses, avoiding misleading answers that may arise from general LLMs when addressing professional medical issues.

Overall, the traditional Chinese medicine case-based Q&A system, based on LLMs and knowledge graphs, provides outstanding performance in terms of security, professionalism, and fluency, significantly outperforming traditional Q&A systems that rely solely on LLMs, and has enormous potential for application.

## 5 Discussion

This study utilizes the *Medical Cases of Wang Zhongqi* as its data source, incorporating a total of 679 cases involving 41 distinct diseases. The research employs LLMs (LLMs) to automatically extract entities from the medical cases, followed by subsequent manual normalization to construct a knowledge graph for TCM cases. By integrating the capabilities of LLMs with the knowledge graph, the study developed a question-answering system for TCM medical cases, successfully enabling natural language queries within the knowledge graph of TCM medical cases. Throughout the research, the natural language understanding and generation capabilities of LLMs, along with the structured representation benefits of knowledge graphs, were fully utilized, offering a novel approach and tool for modern TCM medical case research.

According to the evaluation conducted using the RAGAS assessment framework, the question-answering system integrating LLMs and knowledge graphs achieved scores of 0.9375 in faithfulness, 0.9686 in answer relevance, and 0.9500 in contextual recall, validating the potential and value of the combination of LLMs with knowledge graphs to advance TCM medical case research.

Based on the comprehensive SUS evaluation results, our QA system demonstrates outstanding performance in terms of safety, usability, and smoothness, significantly outperforming other existing QA systems. This demonstrates that the integration of LLMs and knowledge graphs in a TCM medical case-based QA system exhibits remarkable reliability, professionalism, and natural fluency when answering TCM-related questions based on medical case content, highlighting its substantial potential for application.

This paper evaluates the performance of LLMs in automatic Named Entity Recognition (NER) tasks. The results indicate that the number of false positives (FP) exceeds that of false negatives (FN) in most LLMs, suggesting that the model’s recall rate typically surpasses its precision. This phenomenon highlights the unique challenges encountered by Named Entity Recognition in the field of Traditional Chinese Medicine (TCM). The polysemy and specialized nature of TCM terminology are primary factors influencing the accuracy of entity recognition. The same term may have different meanings in various contexts, complicating the entity recognition task. Moreover, TCM-related concepts, such as the names of formulas and herbal ingredients, may not be adequately represented in the training data of LLMs. Although these models can identify common medical terms, their accuracy in recognizing TCM-specific terms and symptoms remains limited. Furthermore, certain traditional expressions in TCM theory, particularly those derived from Classical Chinese, may contribute to an increased number of false positives in the models. This phenomenon primarily arises from the insufficient TCM-specific data available during the training of LLMs. To address this issue, future research could tailor the training of LLMs, particularly by fine-tuning them using TCM literature, case studies, and formula data, to enhance the accuracy of entity recognition in TCM, reduce false positives, and improve precision.

In practical applications of question-answering systems, retrieval failures are occasionally observed, where user queries fail to return relevant case studies or knowledge. Analysis reveals that a significant cause of retrieval failures is the inability to recognize entities in user queries. When the system fails to accurately recognize TCM terms in the query, it cannot extract relevant information from the knowledge base, resulting in empty or inaccurate retrieval results. The root cause of this issue lies in the limited content from the TCM field within the training data of current LLMs. Most LLM training datasets are biased toward modern medicine and other fields, with insufficient support for TCM-specific symptoms, diseases, and treatments. The performance of LLMs is particularly weak when addressing obscure or complex TCM symptoms. To enhance retrieval accuracy, future research could consider fine-tuning LLMs with TCM literature, classical case studies, herbal materials, and formula data to improve their precision in TCM entity recognition.

In conclusion, this study introduces a novel approach and tool for TCM case research by leveraging LLMs to assist in the construction of a knowledge graph for TCM cases and the development of a question-answering system, yielding significant research outcomes. With ongoing technological advancements and the expansion of application scenarios, the integration of LLMs and knowledge graphs is expected to play an increasingly important role in TCM case research, accelerating the exploration of TCM diagnostic and treatment practices.

Due to the variations in diagnostic approaches among various TCM practitioners, the current QA system is primarily based on the *Medical Cases of Wang Zhongqi*. Future work could consider incorporating medical cases from diverse periods and regions to enrich the content and enhance the representativeness of the TCM knowledge graph. Additionally, future efforts will focus on enhancing the intelligence of the QA system and exploring interdisciplinary research and applications. By leveraging the advancements in LLMs, the intelligence of the QA system can be enhanced, enabling it to better understand user needs and provide more accurate responses. This will provide more comprehensive support for clinical diagnosis and treatment.

## Data Availability

The datasets presented in this study can be found in online repositories. The names of the repository/repositories and accession number(s) can be found at: https://github.com/Icheo/TCM-KG-LLM-QASystem.

## Footnotes

^1^ https://pandas.pydata.org/pandas-docs/stable/

^2^ http://py2neo.org/

^3^ https://www.langchain.com/
